# Mortality in hospitalized patients with tuberculous meningitis

**DOI:** 10.1186/s12879-018-3633-4

**Published:** 2019-01-05

**Authors:** Jaime Soria, Tatiana Metcalf, Nicanor Mori, Renee E. Newby, Silvia M. Montano, Luz Huaroto, Eduardo Ticona, Joseph R. Zunt

**Affiliations:** 1 Infectious and Tropical Diseases Department, Hospital Nacional Dos de Mayo, Lima, Peru; 2 Faculty of Tropical Medicine, Mahidol University, Bangkok, Thailand; 3 Universidad Nacional Mayor de San Marcos, Lima, Peru; 4 Faculty of Medicine, Universidad Nacional Mayor de San Marcos, Lima, Peru; 5 Departments of Neurology, Global Health, Medicine and Epidemiology, University of Washington, Seattle, WA USA; 6 Northern Pacific Global Health Research Fellows Training Consortium, University of Washington, Seattle, USA; 7 University of Washington School of Medicine, 1959 NE Pacific St, Seattle, WA 98195 USA; 8 Hospital Nacional Dos de Mayo, Parque Historia de la Medicina Peruana, Av. Grau, 1300 Lima 1, Peru

**Keywords:** Tuberculosis, Meningitis, HIV

## Abstract

**Background:**

To evaluate the mortality in hospitalized patients with tuberculous meningitis and describe factors associated with an increased risk of mortality.

**Methods:**

Retrospective study of hospitalized patients with tuberculous meningitis between 2006 and 2015 in Peru performing a generalized linear regression to identify factors predictive of in-hospital mortality.

**Results:**

Of 263 patients, the median age was 35 years, 72.6% were men, 38% were positive for HIV upon admission, 24% had prior TB infections and 2.3% had prior MDR-TB infections. In-hospital mortality was 30.4% of all study patients with a final diagnosis of TBM. When multivariable analysis was applied, significant associations with in-hospital mortality were seen among patients with HIV (RR 2.06; Confidence Interval 95% (95% CI) 1.44–2.94), BMRC II (RR 1.78; 95% CI 1.07–2.97), BMRC III (RR 3.11; 95% CI 1.78–5.45) and positive CSF cultures (RR 1.95; 95% CI 1.39–2.74).

**Conclusions:**

In-hospital mortality is higher among patients with HIV infections, age over 40 years, positive CSF TB culture and BMRC stage II or III.

## Background

Despite being a preventable and curable disease, tuberculosis (TB) is the leading infectious cause of death worldwide [1]. The World Health Organization estimates that there were 37,000 incident cases of tuberculosis in Peru in 2016 [1]. Peru reported an incidence of 30,988 tuberculosis cases in 2015 of which, 5.9% were multidrug-resistant (MDR) [1]. Approximately 1% of individuals with TB develop involvement of the central nervous system (CNS), which most often manifests as meningitis, tuberculomas or abscesses [2]. Tuberculous meningitis is a severe form of tuberculosis, which is often life-threatening and can produce serious disabilities for those who survive.

HIV infection significantly increases the risk of developing active TB, the rate of progression from latent to active disease, and TB-associated morbidity and mortality [3, 4]. In 2015, an estimated 10.4 million people developed TB and 1.7 million died from the disease including 374,000 deaths from TB among people with HIV [1]. In addition, HIV-infected individuals with TB are five times more likely to develop CNS involvement, which often results in severe morbidity, if not death [3–5].

Symptoms of TB meningitis (TBM) often mimic encephalitis and include fever, headache, vomiting, and altered level of consciousness, although the clinical presentation of TBM is generally similar irrespective of HIV status [5–8]. Some studies have reported impaired consciousness, lymph node involvement, and extra-meningeal TB more frequently in HIV-infected people [3, 5, 9, 10]. Toxoplasma encephalitis, cryptococcal meningitis and TBM are the most common opportunistic CNS infections in patients with HIV infections [11].

The objective of this study was to evaluate the mortality in hospitalized patients with tuberculous meningitis and describe clinical and laboratory features associated with an increased risk of mortality.

## Methods

We carried out a retrospective cohort study, reviewing medical records of adults diagnosed with TB at Hospital Nacional Dos de Mayo (HNDM) in Lima, Peru between 2006 and 2015. HNDM is in a region with a high incidence of tuberculosis in Peru and serves a population of low socio-economic status in Lima. Potential cases were identified by reviewing the database of the hospital’s department of epidemiology and the discharge registry of the infectious diseases department.

We included all cases that met the case definition of definite, probable or possible TBM: Definite TBM required a positive culture for *M. tuberculosis* or acid-fast bacilli (AFB) in cerebrospinal fluid (CSF). Probable TBM was diagnosed in patients with clinical symptoms of meningitis plus isolation of *M. tuberculosis* outside the CNS and exclusion of other potential causes of meningitis. Possible TBM was diagnosed in patients with clinical symptoms of meningitis and CSF findings suggestive of TBM and exclusion of other potential causes, but no isolation of mycobacterium. We excluded patients who had incomplete records, patients under 18 years of age and those who had a final diagnosis other than TBM. We recorded demographic and clinical information, laboratory results, drug treatment and outcome at discharge from the clinical files and microbiology department records. Information related to HIV treatment was obtained from the database of the Peruvian National Antiretroviral Therapy Program.

Standard Ziehl-Neelsen smear was used to detect AFB in CSF; CSF culture was performed using a solid medium (Ogawa modified medium) [12]. Drug susceptibility testing of mycobacterium isolates to second-line drugs was performed at the reference laboratory of the Peruvian Ministry of Health and at the National Institute of Health. CSF macroscopic examination, protein and glucose quantification and cell count were performed at the hospital laboratory. CSF adenosine deaminase (ADA) level was determined according to the package insert (Diazyme, Poway, CA). HIV status was determined using HIV 1 + 2 ELISA test (Wantai, Beijing, China) with a confirmatory immunofluorescent assay (Instituto Nacional de Salud, Lima, Peru). Records of HIV-infected patients were reviewed to obtain results of quantitative HIV viral load and CD4 cell count.

Severity of disease at admission was graded according to the British Medical Research Council (BMRC) criteria: grade I; normal level of consciousness and no focal signs, grade II; lethargy or behavioral changes, meningeal irritation, or minor neurologic deficits such as cranial nerve palsies, and grade III; stupor or coma, abnormal movements or severe neurologic focal deficit.

Descriptive statistics was performed for clinical, epidemiological and laboratory features. Continuous variables were compared using the Student’s t-test or Mann-Whitney U test, and categorical variables were compared by Fisher’s exact test. Shapiro-Wilk test was used to test normality. A log-binomial regression was used to identify variables predictive of in-hospital mortality. Risk Ratio (RR) and 95% CI were reported and variables were tested using a level of significance of 0.05. The statistical analyses were performed with STATA version 12.0 (College Station, Texas).

## Results

Between 2006 and 2015, 3802 patients were admitted with the diagnosis of tuberculosis at HNDM, 1495 (39.3%) of whom had extra-pulmonary TB and 434 (11.4%) had a discharge diagnosis of CNS TB. We located 396 records of patients with a CNS TB diagnosis and excluded 133 records for the following reasons: 37 had a final diagnosis other than TBM, 32 were diagnosed with TBM at another institution and then admitted to HNDM for other reasons, 23 had missing information (e.g. no CSF analysis), 20 were younger than 18-years-old, 14 had focal CNS lesions consistent with tuberculous granulomas without evidence of meningitis and 7 had histopathologic or CSF findings inconsistent with TBM. Overall, 263 adult patients with TBM were identified and included in this analysis. The majority of patients were male (72.6%) and the median age was 35 years (range 18–84 years). Sixty-three patients (24.0%) had a prior history of tuberculosis and 6 (2.3%) had a prior history of MDR-TB. Alcoholism and drug addiction were reported in 60 patients (22.8%) (Table 1).

**Table 1:** Demographic, clinical and CSF characteristics by HIV status

	Total (*n* = 263)	HIV Positive (*n* = 100)	HIV Negative (*n* = 163)	*p*-value
Age (years)^a^	35 (18–84)	35 (18–74)	35 (18–84)	0.899
Male	191 (72.6%)	81 (81.0%)	110 (67.4%)	0.010
TB history	63 (24.0%)	34 (34.0%)	29 (17.8%)	0.002
MDR-TB history	6 (2.28%)	5 (5.0%)	1 (0.3%)	0.030
TB contact	70 (26.7%)	21 (21.0%)	49 (30.1%)	0.116
Alcoholism / Drug addiction	60 (22.8%)	34 (34.0%)	26 (15.9%)	0.001
Symptoms > 7 days	199 (76.1%)	82 (82.0%)	117 (71.8%)	0.075
Weight loss	129 (50.9%)	59 (59.6%)	70 (45.5%)	0.029
Headache	200 (79.1%)	81 (81.0%)	119/154 (77.3%)	0.431
Fever	182 (72.0%)	72 (72.0%)	110/154 (71.4%)	0.886
Altered level of Consciousness	173 (68.1%)	58 (58.0%)	115/155 (74.2%)	0.013
Focal neurologic deficits	91 (36.0%)	31 (31.3%)	60 (38.9%)	0.020
BMRC stage				
I	93 (35.3%)	39 (39.0%)	54 (33.1%)	0.502
II	133 (50.6%)	46 (46.0%)	87 (53.4%)	
III	37 (14.1%)	15 (15.0%)	22 (13.5%)	
WBC^b^ > 10,000 cells/uL	74/253 (29.3%)	15/99 (15.2%)	59/154 (38.3%)	< 0.001
Hyponatremia	146/219 (66.7%)	64/85 (75.3%)	82/134 (61.2%)	0.039
CD4 count^a^		60 (1–874)		
CD4 < 200		64/81 (79.0%)		
Detectable HIV Viral load		76/77 (98.7%)		
CSF Analysis				
Protein^a^	213 (10–6000)	226 (12–1200)	210 (10–6000)	0.550
Glucose^a^	32 (10–118)	33.2 (13.5–82)	31 (10–118)	0.595
Leucocytes^a^	80 (1–3808)	56 (1–1550)	90 (1–3808)	0.040
Mononuclear cells (%)^a^	98 (5–100)	100 (15–100)	95 (5–100)	0.010
ADA^a^	10 (0.8–76.1)	9.8 (0.9–53.3)	10 (0.9–76.1)	0.974

^a^ Median (range), ^b^ White blood cell count

One hundred patients (38.0%) were HIV-infected at the time of diagnosis, 32 (32.0%) of whom were unaware of their HIV positive status prior to admission. Of those with known CD4 cell counts, 64 (79.0%) of 81 had CD4 cell count < 200 cells/mL at the time of TBM diagnosis. Only 18 (18.0%) of HIV-infected patients had started antiretroviral therapy (ART) and 16 (88.9%) of the patients receiving ART had a detectable viral load at the time of TBM diagnosis. HIV-infected patients were significantly more likely than HIV-uninfected patients to have reported TB disease in the past (34.0% vs. 17.8%; *p* = 0.002) and abuse of alcohol or drugs (34.0% vs. 15.9%; *p* = 0.001) (Table 1).

At time of admission, the most common symptoms were headache (79.1%), fever (72.0%) and altered level of consciousness (68.1%). HIV-uninfected patients were significantly more likely to have focal neurological signs (38.9% vs. 31.3%; *p* = 0.020) and altered level of consciousness (74.2% vs. 58.0%; *p* = 0.013). Duration of symptoms varied from a few days to months. 199 (76.1%) reported symptoms for more than 7 days prior to hospital admission. By BMRC clinical staging at admission, 61 (61.0%) HIV-infected patients were at clinical stage II or III, compared to 109 (66.9%) HIV-uninfected patients (*p* **=** 0.502).

Regarding CSF features, pleocytosis (> 5 cells/mm3) was present in 233 (88.6%) and protein level was greater than 45 mg/dL in 227 (92.7%) of 245 patients (Table 1). CSF values for protein, glucose and ADA did not differ significantly between HIV-infected and uninfected patients or in patients with possible, probable or definite MTB. Cell counts were lower in HIV-infected patients with possible MTB than in uninfected patients with possible MTB (*p* = 0.010) (Table 2). CSF ADA was higher in patients with a positive CSF culture (16.2. vs. 10.0; *p* < 0.001), in both HIV-infected and HIV-uninfected patients compared with patients with a negative CSF culture. When comparing CSF among living and deceased patients, there was no significant difference in the level of CSF protein or hypoglycorrhachia, but the median CSF leukocyte count was significantly lower among deceased patients (*p* = 0.027) (Table 3).

**Table 2:** CSF characteristics by HIV status in Possible, Probable and Definite Tuberculous Meningitis

	Possible (*n* = 161)			Probable (*n* = 34)			Definite (*n* = 68)		
CSF Analysis (*n* = 263)	HIV Positive (*n* = 72)	HIV Negative (*n* = 123)	*p*-value	HIV Positive (*n* = 12)	HIV Negative (*n* = 22)	*p*-value	HIV Positive (*n* = 28)	HIV Negative (*n* = 40)	*p*-value
Protein^a^	205 (12–585)	199 (10–2249)	0.891	285 (32–1200)	177 (10–728)	0.092	290 (25–900)	290 (98–6000)	0.773
Glucose^a^	36 (12–78)	36 (10–118)	0.800	33 (21–82)	29 (10–65)	0.347	29 (15–61)	27 (10–89)	0.324
Hypoglycorrhachia	50/54 (92.6%)	80/86 (93.0%)	1.000	7/8 (87.5%)	17/19 (89.5%)	1.000	27/27 (100.0%)	35/35 (100.0%)	1.000
Leukocytes^a^	50 (1–1550)	105 (2–3808)	0.010	45 (1–610)	32 (1–2000)	0.856	82 (1–1165)	83 (4–2160)	0.685
Mononuclear cells (%)^a^	100 (15–100)	95 (5–100)	0.010	100 (20–100)	99 (13–100)	0.241	90 (20–100)	90 (8–100)	0.623
ADA^a^	9 (0.9–32.9)	9 (0.7–52.7)	0.966	11 (2.2–37)	10 (1.9–28.6)	0.524	15 (0.9–53.3)	14 (1–76.1)	0.417

^a^Median (Range)

**Table 3:** CSF features by survival

	Total (*n* = 263)	Surviving (*n* = 183)	Deceased (*n* = 80)	*p*
Protein^a^	213 (10–6000)	207 (10–6000)	245 (19–1200)	0.115
Glucose^a^	32 (10–118)	34 (10–118)	27 (10–111)	0.006
Hypoglycorrhachia^b^	216/229 (94.3%)	146/157 (92.9%)	70/72 (97.2%)	0.610
Leukocytes^a^	80 (1–3808)	90 (1–3808)	60 (1–1140)	0.027
Mononuclear cells (%)^a^	98 (5–100)	98 (5–100)	96 (10–100)	0.650
ADA^a^	10 (0.8–76.1)	9.8 (0.9–52.7)	10.8 (0.8–76.1)	0.251

^a^ Median (range)

^b^ Data are not included for patients without simultaneously drawn CSF and serum glucose quantities

The diagnosis of TBM was confirmed by CSF culture in 68 patients (25.9%): 28 (28.0%) HIV-infected and 40 (24.5%) HIV-uninfected. 70 (35.0%) of 200 had a positive culture in samples from outside the CNS. Of the 68 CNS TB cultures, 45 had drug susceptibility testing, 25 (55.6%) were pan-susceptible and 8 were MDR-TB, with 5/8 (62.5%) in HIV-infected patients versus 3/8 (37.5%) in HIV-uninfected patients (*p* = 0.699). Age over 40 (*p* = 0.002), BMRC class III (*p* < 0.001) and HIV status (*p* < 0.001) were significantly associated with mortality among the 68 definite TB cases.

The treatment administered to 240 patients (91.3%) included a standard combination of four drugs (isoniazid, rifampicin, pyrazinamide and ethambutol); 230 (87.4%) also received steroids; primarily dexamethasone.

Eighty patients (30.4%) died during hospitalization; half within the first 14 days of admission. Death during hospitalization was significantly associated with HIV coinfection, with 42 deaths (52.5%) in HIV-infected and 38 (47.5%) in HIV-uninfected patients (Risk Ratio (RR) 1.80; *p* = 0.001), age > 40 years (RR 1.59; *p* = 0.010), Glasgow coma scale < 14 (RR 2.30; *p* < 0.001), focal neurologic deficits (RR 1.48; *p* = 0.036), BMRC stage II (RR 1.88; *p* = 0.015) or stage III (RR 3.30; *p* < 0.001), increasing ADA value (RR 1.17; *p* = 0.426), and positive culture in CSF (RR 2.11; p < 0.001) (Table 4). Time from hospital admission to treatment (5.1 days vs. 5.0 days; RR 1.0, *p* = 0.850), was not significantly longer for HIV-infected patients than HIV-uninfected patients.

**Table 4:** Factors associated with death during hospitalization

	Surviving (*n* = 183)	Deceased (*n* = 80)	Bivariable Analysis			Multivariable Analysis		
			RR	*95% CI*	*p*	RR	*95% CI*	*p*
Male	134 (73.2%)	57 (71.2%)	0.93	0.63–1.40	0.740	0.79	0.54–1.13	0.201
Age > 40 years old	53 (28.9%)	36 (45%)	1.59	1.12–2.29	0.010	1.64	1.16–2.29	0.004
HIV Infection	58 (31.7%)	42 (52.5%)	1.80	1.25–2.60	0.001	2.06	1.44–2.94	< 0.001
History of tuberculosis	40 (21.9%)	23 (28.8%)	1.28	0.84–1.90	0.217			
Alcoholism / Drug addiction	40 (21.9%)	20 (25.0%)	1.13	0.74–1.71	0.572			
Glasgow < 14	86 (48.9%)	60 (77.3%)	2.30	1.58–3.48	< 0.001	2.08	1.38–2.75	< 0.001
Focal Neurologic deficits	56 (31.8%)	35 (45.5%)	1.48	1.03–2.14	0.036	1.14	0.79–1.64	0.473
BMRC II	90 (49.2%)	43 (53.8%)	1.88	1.13–3.13	0.015	1.78	1.07–2.97	0.027
BMRC III	16 (8.74%)	21 (26.3%)	3.30	1.94–5.59	< 0.001	3.11	1.78–5.45	< 0.001
ADA > 9 UI/L	87 (51.8%)	43 (57.3%)	1.17	0.80–1.71	0.426			
MDR-TB	4 (16.7%)	4 (20.0%)	1.08	0.50–2.39	0.833			
CSF Positive culture	34 (18.6%)	34 (42.5%)	2.11	1.50–3.00	< 0.001	1.95	1.38–2.75	< 0.001
Tuberculosis outside CNS	53 (39.3%)	22 (52.3%)	1.43	0.95–2.15	0.086			

A multivariable model to predict in-hospital mortality was developed using a log-binomial regression. In-hospital mortality was significantly associated with HIV status (RR 2.06; 95% CI1.44–2.94; *p* < 0.001), BMRC II (RR 1.78; 95% CI 1.07–2.97; *p* = 0.027), BMRC III (RR 3.11; 95% CI 1.78–5.45; p < 0.001) and positive CSF culture (RR 1.95; 95% CI 1.39–2.74; *p* < 0.001) (Table 4).

## Discussion

In our study, in-hospital mortality was 30.4% over 10 years, and was highest in patients with HIV infection, age older than 40 years, advanced stage of infection at presentation (BMRC grade II or III) with Glasgow coma scale less than 14 and positive *M. tuberculosis* culture.

Consistent with prior studies, TBM in our study was associated with poor prognosis, especially among HIV-infected patients [5, 8, 13]. Factors previously associated with poor prognosis of TBM in HIV-infected patients have included more severe illness at presentation, CD4 cell count < 50 cells/μL, and presence of MDR-TB [5, 14]. One multicenter cohort study also included diabetes mellitus, hydrocephalus, and vasculitis as prognostic factors (HAMSI scale), and one category included HIV patients with low CD4 counts [15]. Our study confirmed other previously reported findings including no significant differences in CSF characteristics (protein, WBC count and glucose) between patients with and without HIV co-infection, higher likelihood for HIV-infected patients having had extrapulmonary TB, and a higher percentage of extrapulmonary TB presenting as TBM [6, 16]. Although concomitant pulmonary TB was more common in HIV-infected patients (32.6% vs. 29.3%; *p* = 0.4), it was not associated with increased mortality nor with degree of immunosuppression, as measured by CD4 cell count, though it should be taken into consideration that the majority of patients in both the deceased and living groups had CD4 counts less than 200.

Compared to patients without HIV infection, patients co-infected with HIV were significantly less likely to have elevated peripheral WBC counts, altered level of consciousness or neurological deficits, which we postulate is due to the decreased inflammatory response and granuloma formation in HIV-infected patients infected with TB as shown by Orme and Basaraba in 2014 [17]. Nearly all HIV-infected patients, even those who reported receiving antiretroviral therapy, had detectable HIV viral load at the time of diagnosis with TBM, making it unlikely that the immune reconstitution syndrome contributed to higher mortality in our study, this more frequently reflects non-adherence than resistance to ART.

Globally, 4.1% of new cases and 19.0% of previously treated TB cases were estimated to have had MDR-TB in 2016 [1]. People living with HIV infection and MDR-TB infection have extremely high mortality and require prolonged and complicated treatment [13, 18]. Of the 45 *M. tuberculosis* isolates available for susceptibility testing in our study, eight (17.8%) were MDR-TB and of those, five were HIV positive. The majority of patients (91.3%) in our study initiated first-line treatment. Although antimicrobial susceptibility profiles were not available for several weeks after samples were obtained, 20 patients (7.6%) received treatment with second-line drugs due to suspicion of resistance to one or more first-line drugs given their history and risk factors. Three patients did not receive treatment because they died early after hospitalization.

Previous studies have reported previous treatment for tuberculosis, HIV infection, living in urban centers, cavitary pulmonary tuberculosis, highly positive AFB smear as risk factors for MDR-TB [19, 20]. The most common risk factors identified in our study were a history of TB treatment, having finished TB treatment in the last year, a history of more than one episode of tuberculosis, and a history of MDR-TB. In these conditions, some patients received second-line therapy. Standard therapy was prescribed to patients with suspected sensitive TB with no known MDR-TB risk factors. The treatment was modified in cases of MDR-TB after sensitivity results were available – an average of 90 days after admission. Because of this delay and because we were only able to obtain sensitivities for 45 of the patients, it is possible that some cases of MDR-TB were missed which may have led to increased mortality.

Twenty patients in the study started treatment for suspected MDR-TB. Of those, ten had susceptibility testing which showed five with MDR-TB and 5 with rifampicin-sensitive TB. Mortality among those who had MDR-TB was not higher than among those who had sensitive TB during hospitalization. The BMRC stage at time of presentation was not significantly worse for patients with MDR-TB than for patients without MDR-TB.

The main limitation of this retrospective study was the lack of information on long-term outcomes after discharge, largely due to the requirement of Peruvian treatment guidelines requiring that patients receive treatment for TB from health centers within their home district. This limitation prevented us from examining factors associated with long-term morbidity and mortality associated with factors such as HIV co-infection and infection with MDR-TB. Additionally, we were not able to access images for many patients and thus these data were not analyzed. The study did not include children under 18 and we know that these represent many cases in our hospital. The diagnosis of TBM is limited by lack of sensitive diagnostic techniques. Thus, it is possible that additional cases of TBM were not identified. Likewise, testing for MDR-TBM was not performed for all patients and may have played a larger role in mortality than we are aware.

## Conclusions

The association with higher in-hospital mortality stresses the importance of ensuring patients with one or more of the risk factors described in this study for increased mortality receive prompt initiation of therapy. Mortality from TBM during hospitalization was highest in HIV patients who were not receiving ART or did not adhere to ART at time of diagnosis. This study highlights the importance of early detection of HIV and the development of strategies to support adherence to ART particularly in HIV/TB endemic countries in an effort to minimize morbidities and mortalities from concurrent infectious diseases. The study also shows the importance of rapid diagnosis and appropriate treatment in HIV positive patients with suspected tuberculous meningitis who not only will die without proper treatment but will often continue to spread TB due to concurrent pulmonary TB infections.

## Abbreviations

ADA: Adenosine deaminase
AFB: Acid-fast bacilli
ART: Antiretroviral therapy
BMRC: British Medical Research College
CNS: Central nervous system
CSF: Cerebrospinal fluid
ELISA: Enzyme-linked immunosorbent assay
HIV: Human immunodeficiency virus
HNDM: Hospital Nacional Dos de Mayo
MDR: Multi-drug resistant
RR: Risk ratio
TB: Tuberculosis
TBM: Tuberculous meningitis
WBC: White blood cell
